# Occurrence of *Toxoplasma gondii* in raw milk of domestic ruminants and human sera: a seromolecular study from Upper Egypt

**DOI:** 10.1186/s13620-026-00338-2

**Published:** 2026-04-10

**Authors:** Nady Kh. Elbarbary, Ahmed Gareh, Basma Gamal, Marwa O. Abd El-Halim, Mohammed F. Ragab, Ahmed Fotouh, Marwa A. Ali, Yasser M. Mohamed

**Affiliations:** 1 Food Hygiene and Control Department, Faculty of Veterinary Medicine, Aswan University, Aswan, 81528 Egypt; 2 Parasitology Department, Faculty of Veterinary Medicine, Aswan University, Aswan, 81528 Egypt; 3 Department of Food Hygiene and Control (Milk Hygiene), Faculty of Veterinary Medicine, Qena University, Qena, 83523 Egypt; 4 Department of Zoonoses, Faculty of Veterinary Medicine, Benha University, Toukh, 13736 Egypt; 5 Medical Parasitology Department, Faculty of medicine, Damietta University, Damietta, 34517 Egypt; 6 Pathology and Clinical Pathology Department, Faculty of Veterinary Medicine, New Valley University, El-Kharga, Egypt; 7 Micrbiology Department, Faculty of Veterinary Medicine, Aswan University, Aswan, 81528 Egypt; 8 Medical Parasitology Department, Faculty of medicine, Assiut University, Assiut, Egypt

**Keywords:** Food safety, Milk, One health, PCR, Pregnant women, Ruminant, Serology, *Toxoplasma gondii*

## Abstract

*Toxoplasma gondii* is a foodborne protozoan that poses significant health risks to both animals and humans. Raw milk contaminated with *T. gondii* has been identified as a potential infectious source for humans. This cross-sectional study investigated the occurrence of *T. gondii* in raw milk from domestic ruminants and in the serum pregnant women in Aswan, Upper Egypt. A total of 250 milk samples collected from various lactating domestic ruminants and 90 serum samples from pregnant women were examined using ELISA (IgG and IgM) and PCR. Overall, 31.2% of milk samples were seropositive, and 21.6% were PCR-positive. *T. gondii* antibodies were found in 34% of cows, 18% of buffaloes, 38% of ewes, 52% of does, and 14% of she-camels by ELISA, while *T. gondii* DNA was identified in 22% of cows, 8% of buffaloes, 28% of ewes, 42% of does, and 8% of she-camels by PCR. The serum of pregnant women showed that 26.7% were seropositive, and 21.1% had *T. gondii* DNA by PCR. In conclusion, these results demonstrate widespread circulation of *T. gondii* in the study area and highlight raw milk as a potential transmission route. Public health awareness and strict milk pasteurization practices are essential to reduce infection risk, particularly among pregnant women.

**Supplementary Information:**

The online version contains supplementary material available at 10.1186/s13620-026-00338-2.

## Introduction

*Toxoplasma gondii* is a globally distributed zoonotic intracellular protozoan parasite responsible for toxoplasmosis, one of the most common foodborne parasitic infections worldwide. It infects nearly all warm-blooded animals, including humans and livestock [1]. Members of the family Felidae are the only definitive hosts and play a central role in the epidemiology of the disease by shedding environmentally resistant oocysts in their feces. Following sporulation, oocysts can survive for prolonged periods under harsh environmental conditions. In intermediate hosts, including domestic ruminants and humans, the parasite undergoes asexual multiplication, forming tachyzoites during the acute phase and bradyzoites within tissue cysts during chronic infection [2].

It is estimated that approximately one-third of the global human population has been exposed to *T. gondii* [3]. Infection may occur horizontally through the consumption of undercooked meat containing tissue cysts, ingestion of sporulated oocysts from contaminated food or water, or consumption of raw milk contaminated with tachyzoites. Vertical transmission can occur when tachyzoites cross the placenta during acute maternal infection, potentially leading to congenital toxoplasmosis [4]. In pregnant women, primary infection may result in miscarriage, fetal abnormalities, or neonatal complications. In immunocompromised individuals, toxoplasmosis can cause severe and sometimes fatal outcomes [1]. In domestic ruminants, infection is associated with abortion, stillbirth, neonatal mortality, and decreased productivity, leading to substantial economic losses [5].

Although numerous studies in Egypt have reported *T. gondii* exposure in humans and animals [1, 6–11]. However, the current state of toxoplasmosis in Egypt remains uncertain [12]. Antibodies against *T. gondii* have been found in the milk of various animals, including sheep, cattle, buffaloes, camels, goats, and even nursing mothers. Numerous investigations have also connected the intake of unpasteurized milk to human toxoplasmosis infections that can cause symptoms and even death [3].

The clinical manifestations of toxoplasmosis are generally vague and often mimic those of other infections, making clinical diagnosis unreliable without laboratory confirmation. Laboratory diagnosis relies on a combination of biological, histological, serological, and molecular techniques [13]. Serological testing is the most commonly used approach for detecting *T. gondii* exposure in both humans and animals, particularly through the detection of specific antibody classes. Traditional tests include the latex agglutination test (LAT), indirect fluorescent antibody test (IFAT), complement fixation (CF), indirect hemagglutination (IHA), agglutination tests, enzyme-linked immunosorbent assays (ELISA), and modified agglutination test (MAT). Molecular diagnosis using polymerase chain reaction (PCR) for various clinical specimens is also a strong tool for recognizing *T. gondii* DNA due to its high sensitivity and specificity in determining the presence of the parasites in clinical samples, even from minimal biological material such as a single tachyzoites [14].

Accordingly, the present study aimed to investigate the occurrence of *T. gondii* antibodies (IgG and IgM) and parasite DNA, as well as associated risk factors, in raw milk from domestic ruminants and in serum samples from pregnant women in Aswan Governorate, Upper Egypt, using combined ELISA and PCR techniques. By integrating animal and human data, the study sought to evaluate raw milk as a potential route of zoonotic transmission and to provide evidence-based insights relevant to food safety and the One Health framework, which links animal, human, and environmental factors in the control of toxoplasmosis.

## Materials and methods

### Study area

In four districts in the Aswan governorate, Egypt—Edfu (24°58’42.77’’ N and 32°52’32.95’’ E), Kom Ombo (24˚27’8’’ N and 32˚55’42’’ E), Daraw (24°24’24.35” N and 32°55’7.96” E.), and Aswan City (24°5’20.1768’’ N and 32°53’59.3880’’ E)—a cross-sectional analysis was carried out between November 2024 and February 2025. The governorate of Aswan spans a total area of 62,726 km2, of which 12,203 km^2^ are inhabited. It is situated on the east bank of the Nile, close to the first cataract, in southern Egypt, just north of the Aswan Dam. In the summer, the climate of Aswan is extremely hot and arid, with temperatures that may surpass 41 °C. Conversely, the winter is relatively moderate, with an average temperature of 26 °C [15]. According to the latest available statistics, the livestock population in Aswan Governorate, Egypt, is estimated to include approximately 32,185 cows, 10,187 buffaloes, 23,358 sheep, 5,874 goats, and 3,718 camels [16].

### Sample size determination

The required sample size was calculated using the formula described by Thrusfield [17]:

$$n=\frac{{Z}^{2}\times{P}_{exp}\left(1-{P}_{exp}\right)}{{d}^{2}}$$

Where: *n* = required sample, Z = appropriate percentage for the standard deviation at 95% confidence level = 1.96, P_exp_ = expected prevalence of 9.7% [1] for milk samples and 28% for serum samples of pregnant women [9], and d = desired absolute precision (margin of error, standard value of 0.05).

### Sample collection and preparation

#### Milk samples

**Fig. 1:**
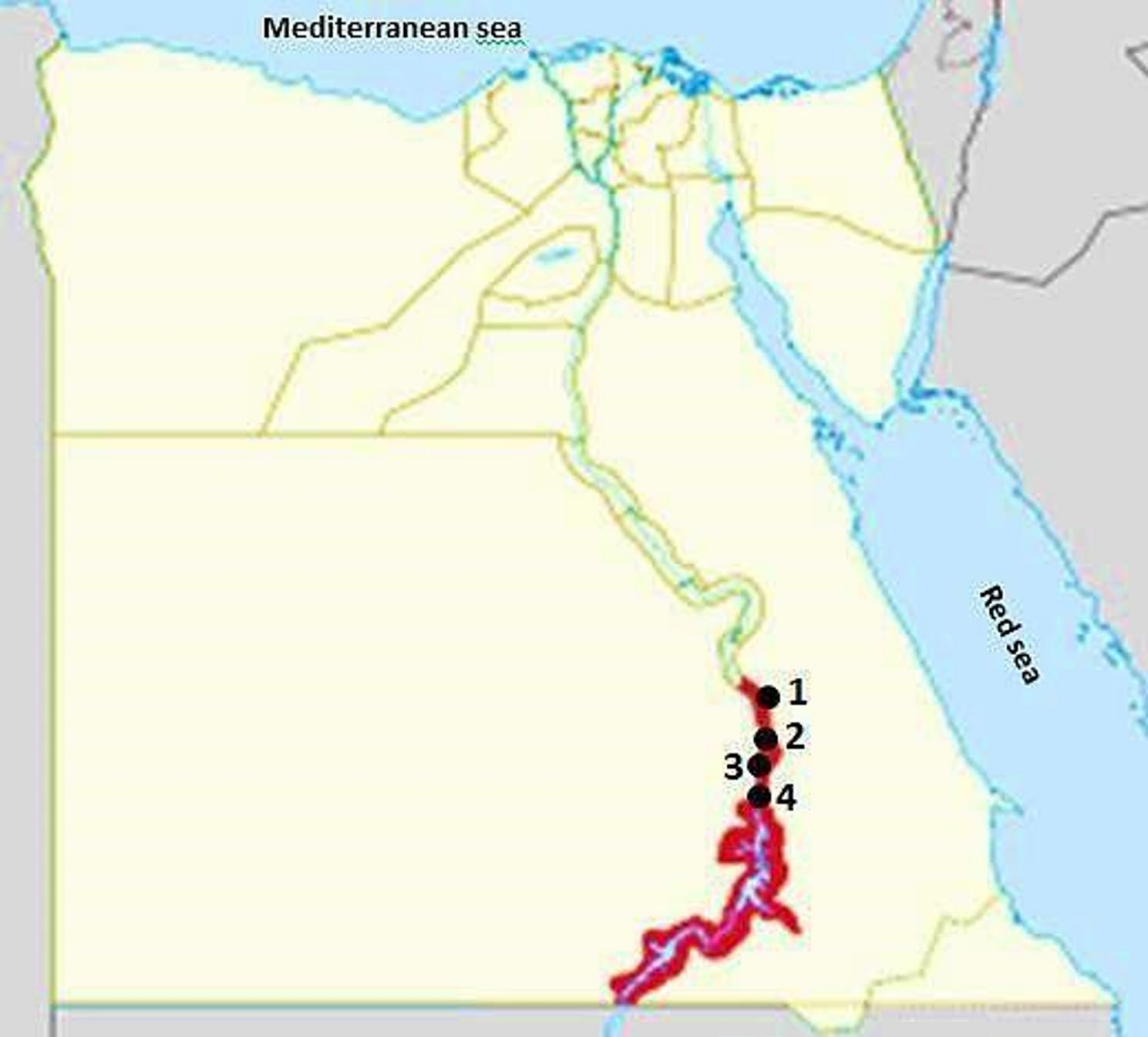
Map of Egypt showing the locations of sample collection. The red area on the map corresponds to the investigated Aswan governorate. Black circles indicate the different cities examined in this study: Edfu (1), Kom Ombo (2), Daraw (3), and Aswan City (4)

A total of 250 raw milk samples were obtained at random from numerous milkings of in-house bred animals: cows, buffalo, ewes, does, and she-camels (50 samples each) in various locations in Aswan Province, Egypt (Fig. 1). Every milk sample (50 ml) was taken by hand from a single animal that seemed to be healthy, using gloves to milk teats that had been cleaned with iodine alcohol. The milk was then put in a sterile screw-cap bottle and sent to the Central Laboratory at the Faculty of Veterinary Medicine at Aswan University and stored at -20 °C until assessment. The milk sample was partitioned into two portions; one portion was centrifuged at 1000× g for 10 min to remove lactoserum from the layer below the lipid layer for the ELISA test. The lactoserum was stored at -20 °C until usage. The second portion remained as whole milk for the PCR test. To estimate the risk factors for toxoplasmosis infection, each animal’s location, sex, age, and breed were recorded.

#### Human samples

Blood samples from the brachial vein of 90 pregnant patients, who were admitted to Obstetrics and Gynecology Department at Aswan University Hospital, were placed in clean, dry, sterile screw-capped vials with labels. Blood samples were divided into two tubes: one was mixed with EDTA for PCR analysis, and the other was allowed to clot at room temperature for 1 h before centrifugation at 1000 ×g for 10 min for the ELISA test. The serum was recovered and stored at -20 °C until analysis. A structured questionnaire was employed by laboratory personnel to obtain data and evaluate risk variables such as age, previous abortion, pregnancy status, duration of current pregnancy, history of *Toxoplasma* infection, consumption of raw milk, and contact with cats.

### ELISA assay

#### Detection of anti-*Toxoplasma gondii* antibodies in milk

The indirect multi-species ELISA assay for *T. gondii* detection (ID.vet, Grabels, France) was employed to detect anti-*T. gondii* antibodies in accordance with the manufacturer’s instructions. The controls were diluted 1:10 and evaluated, while the lactoserum samples were added without dilution [1]. The percentage of sample (S) to positive (P) ratio (S/P %) for each tested sample was computed using the optical density (OD) measurements and the following formula: S/P (%) = (OD sample – OD negative control) / (OD positive control – OD negative control) × 100. If the S/P % was less than 40%, the sample was considered negative; if it was between 40% and 50%, it was considered dubious; and if it was more than 50%, the test was considered positive. The optical density of all ELISA data was determined at 450 nm using an Infinite^®^ F50/Robotic ELISA reader (Tecan Group Ltd., Männedorf, Switzerland).

#### Detection of anti-*Toxoplasma gondii* antibodies in the serum of pregnant women

Commercially available ELISA kits (DRG^®^ *Toxoplasma* IgM (TORCH) (EIA-1799), DRG International, Inc., USA) and (DRG^®^ *Toxoplasma* IgG (TORCH) (EIA-1798), DRG International, Inc., USA) were used to evaluate serum samples for anti-*T. gondii* IgG & IgM antibodies in accordance with the directions provided by the manufacturer. For IgG & IgM detection, results were interpreted using the T. gondii index value provided by the kit. Samples were considered positive when the index was ≥ 1.0 (> 32 IU/mL), harmful when the index was < 0.90 (< 32 IU/mL), and equivocal when the index ranged between 0.91 and 0.99. Equivocal samples were retested to confirm the final result.

### PCR assay

#### DNA extraction and preparation

The PCR technique was employed to assess milk and human blood samples from the animals. DNA was extracted from the whole blood samples and bulk milk samples by using the Quick-gDNA™ MiniPrep kit (Cat. No. D3024, Zymoresearch, USA). Following the manufacturer’s instructions, about a 0.5 mL blood of human with EDTA, as well as 25 mg of the pellet obtained from 10 mL of milk samples subjected to centrifugation at 14,000 rpm/2 min, the creamy and lactoserum layers were carefully removed. The extracted DNA samples were subjected to quantification by the NanoDrop device (NanoDrop, Thermo Scientific, Waltham, USA) [18].

#### Nested Polymerase Chain Reaction (nPCR)

nPCR was employed to further increase the specificity of amplification by targeting an internal sequence of the *T. gondii B1* gene. This step ensured that faint or borderline bands from the first round could be verified and minimized the risk of false positives due to non-specific amplification. The *B1* gene of *T. gondii* was detected through conventional nPCR using a specific primer sequence (W1020300X, Willowfort Co., UK) listed in Table 1 [19]. For the first round of PCR, the following were assembled: 0.5 µM primers, 200 µM dNTPs, 1.5 mM MgCl₂, 1.5 units Amplitaq polymerase, and 10 µL DNA template were used, with a total volume of 50 µL. Similar to the first round, the second round’s amplification reaction employed 10 µL of the previous round’s PCR product as a template for a total volume of 50 µL. Locally derived isolates from the Animal Health Research Institute, Egypt, served as positive controls. The ddH₂O was used for the negative control. The PCR yield was placed on a 3% agarose gel with SYBR Safe DNA gel dye (Invitrogen, USA), run at 100 V for 30 min, and visualized using a transilluminator (GEL DOC XR, Bio-Rad Laboratories, Hercules, CA, USA).

**Table 1:** Primers and settings for PCR recognition of *T. gondii B1* gene

Primer	Sequence (5′- 3′)	Size	PCR conditions
First round (Outer primer)	F: GGAACTGCATCCGTTCATGAG	200 bp	95 °C for 5 min followed by 40 cycles (95 °C / 30 s, 56 °C / 30 s, 72 °C/30 sec)
	R: TCTTTAAAGCGTTCGTGGTC		
Second round (Inner primer)	F: TGCATAGGTTGCAGTCACTG	100 bp	95 °C for 5 min followed by 35 cycles (95 °C / 30 s, 56 °C / 30 s, 72 °C / 30 s)
	R: GCGACCAATCTGCGAATACACC		

### Statistical analysis

SPSS version 16.0 was used for the statistical study, and the chi-square test (χ^2^) was employed to examine the correlation between the toxoplasmosis infection and each of the variables, such as age, sex, and location. *p* < 0.05 was regarded as the significant level.

## Results

### Seroprevalence and risk factor assessment of *T. gondii* seropositivity in the studied animals

Out of 250 raw milk samples of various ruminants examined, 78 (31.2%) had *T. gondii* antibodies using a commercial ELISA kit. *T. gondii* antibodies were identified in 34% (17/50) of cows, 18% (9/50) of buffaloes, 38% (19/50) of ewes, 52% (26/50) of does, and 14% (7/50) of she-camels, respectively. In addition, a statistically significant variance (χ^2^: 11.72; *p* = 0.019) was observed between the different species (Table 2).

**Table 2:** Seroprevalence and risk factors related to *T. gondii* in raw milk samples of tested animals

Variable	Category	Examined no.	Positives no.	Seroprevalence (%)	*p*-value	χ^2^
Total		250	78	31.2		
Species	Cow	50	17	34	0.019^*^	11.72
	Buffalo	50	9	18		
	Ewe	50	19	38		
	Doe	50	26	52		
	She-camel	50	7	14		
Age	˂ 2 years	65	16	24.6	0.416	1.75
	2–5 years	68	19	28.0		
	> 5 years	117	43	36.8		
Breed	Local	166	60	36.1	0.079	3.07
	Mixed	84	18	21.4		
Region	Aswan city	64	13	20.3	0.189	4.77
	Daraw	37	9	24.3		
	Kom Ombo	77	25	32.5		
	Edfu	72	31	43.1		

^*^The result is significant at *p* < 0.05

The seropositivity prevalence of *T. gondii* in relation to age, breed, and regions is displayed in Table 2. No statistically significant relationship existed between the seropositive rate and the prevalence of anti-*T. gondii* antibodies in this set of variables (*p* > 0.05).

The prevalence of anti-*T. gondii* antibodies exhibited a higher level of seropositivity in animals > 5 years old (36.8%) and animals between 2 and 5 years old (28%) compared to animals ˂5 years old (24.6%) (χ^2^: 1.75; *p* = 0.416). Regarding animal breeds, local breed animals exhibited a higher seroprevalence for *T. gondii* (36.1%) than mixed breed animals (21.4%) (χ^2^: 3.07; *p* = 0.079).

Geographical location was not significantly associated with *T. gondii* seropositivity (*p* = 0.189). Although animals from Edfu (43.1%), Kom Ombo (32.5%), and Daraw (24.3%) showed numerically higher prevalence rates compared to Aswan City (20.3%), these differences were not statistically significant (χ^2^: 4.77; *p* = 0.189). 

### Analysis of *T. gondii* seropositivity in pregnant women’s serum: seroprevalence and risk factors

Table 3 shows that a total of 26.7% (24/90) of pregnant women tested positive for *T. gondii* antibodies. Among the seropositive cases, 5.6% (5/90) were IgM-positive, indicating recent infection, while 21.1% (19/90) were IgG-positive, consistent with past or chronic exposure. A statistically significant difference was observed between IgG and IgM detection rates (χ² = 7.24; *p* = 0.007). Regarding demographic variables, pregnant women over 25 years showed a higher seroprevalence (28.1%) than pregnant women under 25 years (23.1%); however, this difference was not statistically significant (χ^2^: 0.142; *p* = 0.7061).

**Table 3:** Seroprevalence and risk factors associated with *T. gondii* in pregnant women serum

Variable	Category	Examined no.	Positives no.	Seroprevalence (%)	*p*-value	χ^2^
Total		90	24	26.7		
Antibodies	IgG	90	19	21.1	0.007^*^	7.24
	IgM		5	5.6		
Age	˂ 25	26	6	23.1	0.706	0.142
	˃ 25	64	18	28.1		
Residence	Urban	22	4	18.2	0.419	0.651
	Rural	68	20	29.4		
Abortion history	None	24	4	16.7	0.354	2.074
	1st time	38	9	23.7		
	More than one time	28	11	39.3		
Contact with cat	Yes	48	19	39.6	0. 022^*^	5.219
	No	42	5	11.9		
Consumption of raw milk	Yes	19	10	52.6	0.039^*^	4.221
	No	71	14	19.7		

^*^The result is significant at *p* < 0.05

Regarding residence, individuals living in rural areas showed a higher seroprevalence for *T. gondii* (29.4%) compared to those living in urban areas (18.2%) but the difference did not reach statistical significance (χ^2^: 0.651; *p* = 0.419). With respect to obstetric history, women with more than one previous abortion demonstrated higher seroprevalence (39.3%) compared to those with a first abortion (23.7%) and those with no history of abortion (16.7%); nevertheless, the association was not statistically significant (χ^2^: 2.074; *p* = 0.354).

In contrast, significant associations were observed for contact with cats (χ^2^: 5.219; *p* = 0.022) and raw milk consumption (χ^2^: 4.221; *p* = 0.039). Pregnant women reporting cat contact had a significantly higher seroprevalence (39.6%) than those without cat contact (11.9%). Likewise, women who consumed raw milk exhibited markedly higher seropositivity (52.6%) than those who consumed boiled or pasteurized milk (19.7%).

### Molecular detection of *T. gondii* using nPCR

**Fig. 2:**
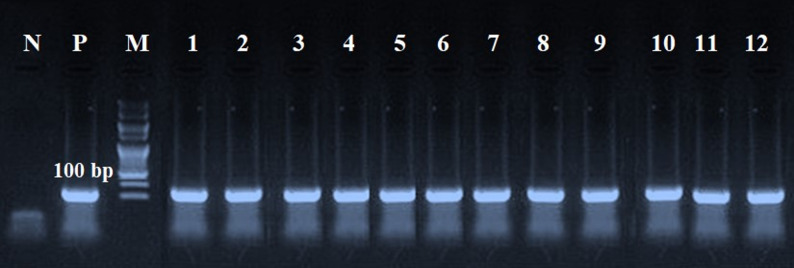
Nested polymerase chain reaction amplification for the detection of *Toxoplasma gondii* in ruminants’ milk samples and women’s sera. Second round targeted to the *B1* gene of *T. gondii* at 100 bp. M: DNA ladder; P: positive control; N: negative control. Lanes 1–2: cows, lanes 3–4: buffaloes, lanes 5–6: ewes, lanes 7–8: does, lanes 9–10: she-camels, and lanes 11–12: pregnant women’s serum

In most positive samples, amplification products were already detectable after the first round of PCR at 200 bp (First-round PCR gels are available in Supplementary Fig. S1). Consequently, the banding patterns between the first and second PCR rounds appear similar in some gel images. However, the nested PCR at 100 bp confirmed specificity and served as an additional safeguard, particularly for samples with faint signals. DNA was detected in milk and pregnant women’s serum using nPCR targeted at the *B1* gene of *T. gondii* with a rate of 21.5% (73/340) (Fig. 2).

### Comparison between ELISA and nPCR

Table 4 summarizes the comparative results of ELISA and nPCR for *T. gondii* detection. Serological analysis demonstrated an overall positivity rate of 30.0% (102/340), whereas molecular detection by nPCR identified parasite DNA in 21.5% (73/340) of the examined samples.

**Table 4:** Prevalence of *T. gondii* antibodies and DNA in raw animals’ milk and pregnant women serum samples using ELISA and nPCR

Category	Samples	Examined no.	Seroprevalence no. (%)	nPCR positives no. (%)
Cow	Milk	50	17 (34)	11 (22)
Buffalo		50	9 (18)	4 (8.0)
Ewe		50	19 (38)	14 (28)
Doe		50	26 (52)	21 (42)
She-camel		50	7 (14)	4 (8.0)
Sub-total		250	78 (31.2)	54 (21.6)
Pregnant women	Serum	90	24 (26.7)	19 (21.1)
Total		340	102 (30)	73 (21.5)

All PCR-positive samples were seropositive, and no PCR-positive/seronegative cases were observed. This indicates that molecular detection was confined to individuals or animals with detectable antibody responses, suggesting that PCR-confirmed infections occurred in cases with established serological evidence of exposure. These findings reflect a strong concordance between serological and molecular methods in the present study.

Agreement analysis demonstrated substantial concordance between ELISA and nPCR (Cohen’s κ = 0.78). The observed agreement was 91.5%. McNemar’s test revealed a statistically significant difference between the two diagnostic methods (χ² = 29.0; *p* < 0.001), with ELISA identifying significantly more positive cases than nPCR.

## Discussion

In the present study, a cross-sectional study design was used to evaluate the prevalence and associated risk factors of *T. gondii* antibodies in raw milk collected from different ruminant species in Aswan Governorate, Upper Egypt. Several serological assays are available for the detection of *T. gondii* infection; however, ELISA is the most widely used method because of its high sensitivity, specificity, rapid processing, and applicability for large-scale epidemiological investigations [20, 21]. The current research serologically found that *T. gondii* was present in 31.2% of the milk of the investigated animals, with ewes and does having a significantly higher percentage than other species. Our investigation has shown that the seroprevalence for *T. gondii* in raw milk in cows (34%) matched that recognized in Pakistan (38%) by Khan et al. [22] and was higher than that of Asiyabi et al. [23] in Iran (3.63%), Fereig et al. [24] in Egypt (2.4%), Fereig et al. [1] in Egypt (9.7%), and Liu et al. [25] in China (6.4%) but lower than that earlier documented in Egypt (64%) by Mohamed et al. [9]. Additionally, the seroprevalence of *T. gondii* in raw milk from buffaloes (18%) was higher than that reported by Asiyabi et al. [23] in Iran (3.3%) but lower than that of Khan et al. [22] in Pakistan (42.1%). In contrast, Fereig et al. [24] failed to detect any *T. gondii* antibodies in buffalo milk. Our results in ewes (38%) matched those identified by Mohamed et al. [9] in Egypt (34%) and were lower than those of Fereig et al. [24] in Egypt (66.7%), Khan et al. [22] in Pakistan (65.8%), and Saad et al. [11] in Egypt (60%).

In the case of does, our results (52%) were consistent with those identified by Mohamed et al. [9] in Egypt (60%) and Gazzonis et al. [26] in Italy (63.3%) and higher than that of Dahmane et al. [3] in Algeria (48.1%) and Liu et al. [25] in China (9.7%) but lower than that recorded in Pakistan (65.2%) by Khan et al. [22] and in Egypt (81.8% and 90%) by Fereig et al. [24] and Saad et al. [11]. Regarding the results of she-camel samples (14%), lower values of 3.3% were obtained by Alipour et al. [27] in Iran and Saad et al. [11] in Egypt, but a higher prevalence of 13.3% was recorded in Iran by Asiyabi et al. [23]. The variation in these findings may be attributed to the infection phase, lactation stage, immunological status at the time of sampling, cat population in the research area, sample size, unsanitary conditions, animal handling, polluted water containing oocysts, and the type of serological assays used.

Previous findings suggested that identifying antibodies in raw milk samples is a more viable and non-invasive option than evaluating serum samples for anti-*T. gondii* antibodies [24]. Thus, using milk provides several advantages: it is easier and less expensive; additionally, milk sample collection reduces the incidence of unintended needle-transmitted infections and minimizes stress-related productivity losses [1]. Another public health advantage of testing milk samples for *T. gondii* is that milk represents both a potential transmission route and a practical, non-invasive surveillance material. The detection of *T. gondii* antibodies or DNA in milk indicates exposure or infection in lactating animals. It highlights the possible risk of parasite transmission to humans through the consumption of raw or unpasteurized milk [22]. However, using milk samples for epidemiological surveillance has inherent limitations. Only lactating animals can be included in such investigations, meaning that non-lactating, young, male, dry, or severely ill animals are excluded. As a result, milk-based screening may underestimate the true prevalence of infection within the entire animal population. Therefore, milk testing should be considered a complementary surveillance tool rather than a comprehensive indicator of herd-level infection [24].

However, natural amounts of immunoglobulins in animal milk throughout lactation may result in varied prevalence in milk samples, necessitating more analysis. Thus, molecular techniques are employed to validate the presence of *T. gondii* DNA in samples. According to our research, *T. gondii* DNA was found in 21.6% (54/340) of raw milk samples from different ruminants. Previous research has confirmed the presence of *T. gondii* DNA in milk samples from various animal species, including cows, buffaloes, does, ewes, and she-camels [11, 22, 24, 28]. Raw milk may contribute to the maintenance of *T. gondii* infection within animal herds through transmission to suckling offspring, and its consumption without proper heat treatment may increase the risk of infection in humans [20].

Our investigation found DNA of *T. gondii* in raw milk samples from 22% of cows, 8% of buffaloes, 28% of ewes, 42% of does, and 8% of she-camels. Furthermore, all animals with *T. gondii* DNA detected by PCR were also seropositive, and no cases of PCR positivity with seronegativity were identified. These results demonstrate substantial concordance between ELISA and nPCR in the tested ruminant species. Nevertheless, the presence of seropositive/PCR-negative cases indicates partial discordance, likely reflecting differences in the detection targets: serology identifies prior exposure, whereas PCR detects circulating parasite DNA.

The present findings suggest that parasite DNA in milk was detected only in animals with detectable antibody responses. However, this observation should be interpreted with caution, as the absence of PCR positivity in seronegative animals does not necessarily exclude early infection stages. Differences in parasite load, intermittent DNA shedding, timing of sampling, and methodological sensitivity may influence molecular detection [24]. Previous studies have reported similar findings, with PCR positivity largely confined to seropositive animals, supporting the association between active infection and an established immune response [3]. In contrast, other investigations have detected parasite DNA in seronegative animals, possibly reflecting very early infection before seroconversion or transient parasitemia. These discrepancies highlight the complexity of *T. gondii* infection dynamics and the importance of combining serological and molecular methods for comprehensive epidemiological assessment [22].

In addition, the lower prevalence of *T. gondii* DNA identified by PCR compared to anti-*T. gondii* antibodies is due to the fact that IgG antibodies develop late in the infection, and the parasite is restricted in the organs and tissues rather than circulated in the blood and eventually reaches the milk [20]. Additionally, the identification of DNA in milk corresponds with the stage of parasitemia, which is short-term and transient in the studied parasite, compared to the long-lasting antibody response [24]. These findings were higher than those found by Saad et al. [11] in Upper Egypt, who detected *T. gondii* DNA in raw milk from does, ewes, and she-camels at rates of 3.7%, 5.6%, and 0%, respectively. However, Dahmane et al. [3] in Algeria found *T. gondii* DNA in 25.5% of the seropositive milk samples from does. Additionally, *T. gondii* DNA positivity in milk has been identified by Khan et al. [22] to be 14.5%, 34.8%, 20%, and 26.3% in ewes, does, cows, and buffaloes, respectively. These findings supported the findings of Fereig et al. [24], who demonstrated that samples from ewes and does with significant antibody reactivity, as determined by ELISA, had a positive PCR outcome for the *B1* gene of *T. gondii*.

Risk factor analysis demonstrated that animal species was significantly associated with *T. gondii* infection. Comparing animal species, it was found that does and ewes had a higher risk of *T. gondii* infection than cows and buffaloes. The current high seropositivity rate in does and ewes indicates that they are very susceptible to *T. gondii* infection and are more likely to come into contact with the parasite. This could be because they have access to polluted pastures and water sources [3]. However, does have a higher seroprevalence of *T. gondii* than ewes. This difference is explained by the fact that does are more susceptible to *T. gondii* infection, due to their grazing habits and higher level of activity and movement compared to ewes, all of which increase the chances of coming into contact with contaminants [9].

Furthermore, age, breed, and region were examined as risk factors for *T. gondii* seropositivity in the present study. However, none of these factors had a substantial impact on *T. gondii* seropositivity. The present investigation found that adult animals had a higher prevalence of *T. gondii* infection in their milk compared to young animals. The observed difference may be due to the fact that animals reared for dairy production and reproduction often lives significantly longer. Animals with longer life expectancies are more likely to be exposed to risk factors, which mean that their milk is more likely to be contaminated with the parasite [29]. The higher seroprevalence observed in older animals may reflect cumulative exposure over time rather than reduced innate resistance. Age-related differences in *T. gondii* infection are complex. Young animals may be more susceptible due to an immature immune system, whereas older animals may exhibit higher antibody prevalence as a consequence of prolonged environmental exposure. In addition, increased vulnerability in older animals may occur under conditions of stress, pregnancy, or immunosuppression rather than solely as a result of aging. Therefore, age-related variation in seropositivity likely reflects differences in exposure history and immune dynamics rather than simple differences in disease resistance [30]. The current study found that native breeds have a higher infection rate than imported breeds, likely due to uncontrolled movements of livestock and shared grazing areas for local breeds, as well as differences in their susceptibility to *T. gondii* infection [31].

Although geographical location was not statistically associated with *T. gondii* seropositivity in the present study, a numerically higher prevalence was observed in the Edfu district. This variation may relate to environmental conditions such as higher temperature and humidity, which can favor oocyst survival, as well as differences in animal management practices and stray cat density [7, 20]. Similar environmental and ecological factors have been discussed in previous studies conducted by Fereig et al. [24] in Egypt, Gharekhani et al. [28] in Iran, Liu et al. [25] in China, Stelzer et al. [32] in Germany, and Wannapong et al. [2] in Thailand, where climatic conditions and cat population density were suggested as contributors to regional variation in infection rates. The results of this investigation indicate that raw milk samples from several ruminant species are suitable for tracking *T. gondii* antibodies. According to the present investigation, the existence of recent *Toxoplasma* infections in animals may be a major source of infection transmission to humans. A significant association between *T. gondii* seroprevalence in pregnant women and the intake of polluted raw milk in Egypt has been demonstrated by Ibrahim et al. [33] and Mohamed et al. [9].

In this investigation, IgG and IgM seropositivity were demonstrated in 21.1% and 5.6% of the 90 pregnant females examined for *T. gondii*, respectively, resulting in an overall prevalence rate of 26.7%. The IgM antibody is the first immunoglobulin to be produced in response to an immunogenic agent. As such, it serves as a reliable indicator of recent infection, which can aid in the identification of acute illness. Typically, detectable IgM is observed approximately one week after exposure to *T. gondii*, and its levels then increase, reaching a maximum after one to three months. In such instances, positive IgM findings should be verified by further specific serological analyses, such as measurement of the IgG antibody (indicating chronic infection) and/or molecular analysis [34]. However, *T. gondii* DNA was found in 21.1% of the sera of pregnant women tested. This result demonstrated the high prevalence of *T. gondii* infection in the research area.

Closely related results were obtained by Mohamed et al. [9], who indicated that 26% and 2% of pregnant women’s serum tested showed seropositivity to IgG and IgM, respectively. Furthermore, the present findings are supported by those of Khan et al. [22] in Pakistan, who reported that 12.4% and 10.9% of female serum tests exhibited seropositivity to IgG and IgM, respectively, resulting in a total prevalence rate of 23.4%. Similarly, Abdelbaset et al. [35] found that pregnant women in El-Minya, Egypt, had an overall seroprevalence of 22.9%. Our findings are lower than those published by Laboudi et al. [36] in Morocco (43%), Robinson et al. [37] in France (31.3%), and Olariu et al. [38] in Romania (55.8%), but higher than those reported by Fanigliulo et al. [39] in Italy (13.8%) and Sebaa et al. [34] in Algeria (13.6%). Both globally and domestically, the prevalence rate of *T. gondii* infection varies because it is influenced by environmental factors (water quality, sanitation, coverage, etc.), socioeconomic factors (hygiene, food source, etc.), climate variables (humidity, temperature), local lifestyle factors (dietary habits, cooking methods, hand washing, type of food item cleaning, contact with cats and other domestic animals, contact with the soil, etc.), and differences in serological analyses with variable specificity and sensitivity [22, 30].

Additionally, cats are commonly kept in or near households, and transmission may be facilitated by poor hygiene practices and limited awareness [7]. In the present study, women aged 25 years or older showed a slightly higher seroprevalence of *T. gondii* antibodies (28.1%) than women younger than 25 years (23.1%); however, this difference was not statistically significant (*p* > 0.05). Therefore, age could not be confirmed as an independent risk factor in this dataset. The observed numerical increase may reflect cumulative exposure over time and the persistence of IgG antibodies for years after infection.

The current investigation also demonstrated that women who have undergone more than one abortion have higher levels of *T. gondii* antibodies than those who have never undergone one or have no history of abortion. This phenomenon may be linked to a hormonal disturbance or immunological suppression [34]. The conclusions here speculated are also supported by findings published elsewhere [9, 12, 22, 34, 37, 39]. These results suggest that consuming or handling raw contaminated milk may increase the risk of *T. gondii* infection in pregnant women. Additionally, the practice of consuming some traditional Egyptian milk products, which are commonly referred to as Karish cheese and are produced from raw bovine milk, may add to and worsen the risk of contracting toxoplasmosis in the community that was under investigation.

This study has several limitations. First, it represents a regional screening of selected lactating animals and pregnant women and may not reflect the true prevalence in the entire animal or human populations of Upper Egypt. Second, the current study did not investigate other well-established routes of infection, such as ingestion of undercooked meat, exposure to contaminated soil, consumption of unwashed vegetables, or drinking contaminated water. Therefore, the relative contribution of raw milk compared with these other transmission routes cannot be determined. Third, detailed geographical provenience and lifetime residence of the pregnant women were not available, limiting the ability to associate exposure with the current study area. These limitations restrict the ability to draw causal inferences, and the findings should be interpreted with caution. Finally, molecular characterization of *T. gondii* isolates through DNA sequencing and phylogenetic analysis was not performed. As a result, circulating strain types and their potential virulence profiles remain undetermined. Moreover, the study population was limited to pregnant women and lactating animals, and other high-risk human or animal groups were not included; these will be taken into account in future investigations.

Despite these limitations, the study provides important baseline data on the circulation of *T. gondii* in animals and humans within a One Health framework. Future investigations with larger sample sizes, expanded geographic coverage, detailed exposure assessment, multivariate modeling, and molecular genotyping are recommended better to understand transmission dynamics and their public health impact.

## Conclusions

The current research revealed the presence of *T. gondii* antibodies and parasite DNA in milk from domestic ruminants, suggesting a potential risk of its transmission to humans through the consumption of unprocessed, unpasteurized milk. The combined use of serological (IgG/IgM) and molecular (PCR) techniques provided a more comprehensive understanding of infection dynamics. Detection of IgG antibodies indicated widespread prior exposure, while IgM positivity and PCR detection suggested recent or active infection in both animals and humans. The study of risk factors associated with the infection provided timely and relevant information for public health and food safety. The data collected supported the potential of milk to be used in the screening of *T. gondii* infection and suggested that raw milk may be involved in the transmission of this protozoan to humans. These findings underscore the need for greater public health awareness of safe milk consumption practices. Pasteurization or proper boiling of milk remains an effective preventive measure to eliminate the parasite. Additionally, integrated One Health control strategies—including improved farm hygiene, control of stray cat populations, routine screening of dairy animals, and monitoring of high-risk human groups—are essential to reduce *T. gondii* transmission in the study area. 

## Supplementary Information

Supplementary Material 1.

## Data Availability

The manuscript included all our available data.

## Abbreviations

OD: Optical density
χ2: Chi-square test
