# Supplementary material for: Occurrence of Toxoplasma gondii in raw milk of domestic ruminants and human sera: a seromolecular study from Upper Egypt
Source: Ir Vet J. 2026 Apr 10;79:22. doi: 10.1186/s13620-026-00338-2 (PMC13077921; doi:10.1186/s13620-026-00338-2)
Supplement: Supplementary file 1 — Supplementary Material 1. [file 13620_2026_338_MOESM1_ESM.docx]

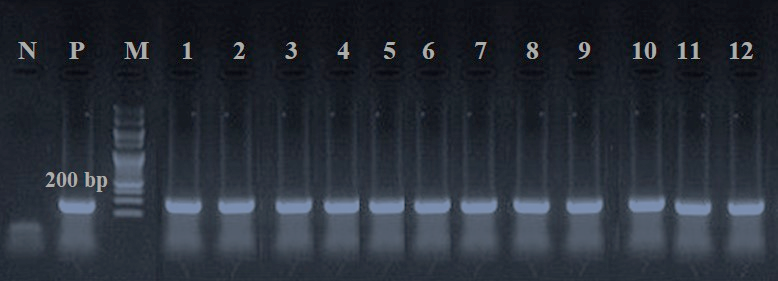


**Supplementary Fig. S1:** The first round of PCR amplification uses the outer pair of primers targeted to the *B1* gene of *T. gondii* at 200 bp. M: DNA ladder; P: positive control; N: negative control. Lanes 1-2: cows, Lanes 3-4: buffaloes, Lanes 5-6: ewes, Lanes 7-8: does, Lanes 9-10: she-camels, and Lanes 11-12: pregnant women's serum.
